# 103. Evaluating The Appropriateness of Vaccine Administration in Patients with Splenectomy

**DOI:** 10.1093/ofid/ofac492.181

**Published:** 2022-12-15

**Authors:** Barbara Barsoum, Anan Rayn, Martin Gozar, Sumeet Jain, Lawrence Ha, Henry Donaghy, Thien-Ly Doan

**Affiliations:** Long Island Jewish Medical Center, New Hyde Park, New York; Long Island Jewish Medical Center, New Hyde Park, New York; Northwell Health, Manhasset, New York; North Shore University Hospital, Westbury, New York; North Shore University Hospital, Westbury, New York; Northwell Health, Manhasset, New York; Long Island Jewish Medical Center, New Hyde Park, New York

## Abstract

**Background:**

The spleen removes microorganisms from the bloodstream and produces antibodies for enhanced immune response. Patients with asplenia are at increased risk for infections caused by encapsulated organisms and have a 6-fold increased risk of sepsis compared to the general population. Those that acquire an infection have a mortality rate of 80%. The CDC recommends administering the meningococcal, influenza, pneumococcal, and Haemophilus influenzae type B (Hib) vaccine at least 2 weeks before or after splenectomy.

**Methods:**

This retrospective chart review evaluated the appropriateness of vaccine administration for patients undergoing splenectomy at Long Island Jewish Medical Center (LIJMC) from January 2016 to June 2020. Patients were included if they were admitted to LIJMC, 18 years or older, and had splenectomy at LIJMC within previous 7 years. Patients were excluded if they were pregnant, admitted to an outside hospital for splenectomy, or had an unknown time of splenectomy. The primary objective was appropriateness of vaccine administration. Inappropriateness was defined as having at least 1 error with administration, including vaccine omissions, timing of vaccine, sequencing (Pneumovax®23 given before Prevnar13®), vaccine interaction (Menactra® given with Prevnar13®), or duplicated vaccines.

**Results:**

Of the 174 patients screened, 29 patients were included in analysis, with a mean age of 58.2 ± 20.6 years. The splenectomy was elective in 69% of patients and emergent in 31% of patients. The vaccine regimen was given inappropriately in 96.6% of patients. The reasons for inappropriateness were vaccine selection (100%), timing (39.3%), vaccine sequence (39.3%), vaccine interaction (14.3%), and duplicate vaccines (7.1%). Excluding emergent splenectomies, 100% of patients received an inappropriate vaccine regimen. The vaccines were given at least 2 weeks before elective splenectomies in 40% of patients, peri-operatively in 30% of patients, and not given in 25% of patients.

**Figure:**
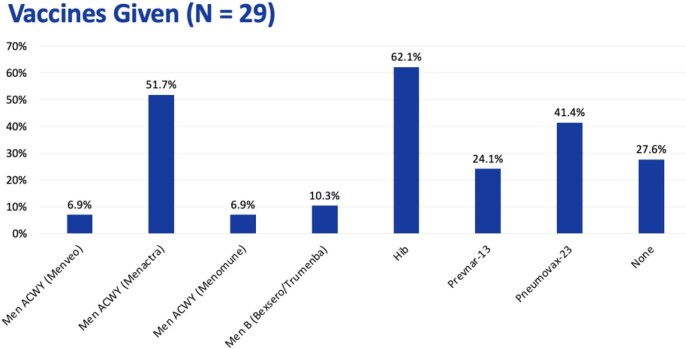

**Figure:**
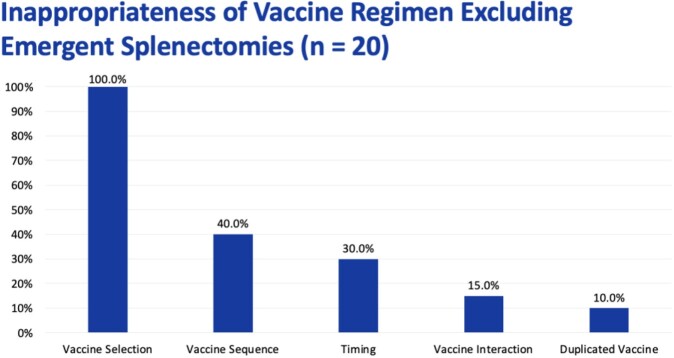

**Conclusion:**

Patients undergoing splenectomy received inappropriate vaccine regimens mainly due to omissions, timing, and sequencing. Prevnar13® should be given before Pneumovax®23 when both are indicated and vaccines should be administered at least 2 weeks before splenectomy.

**Figure:**
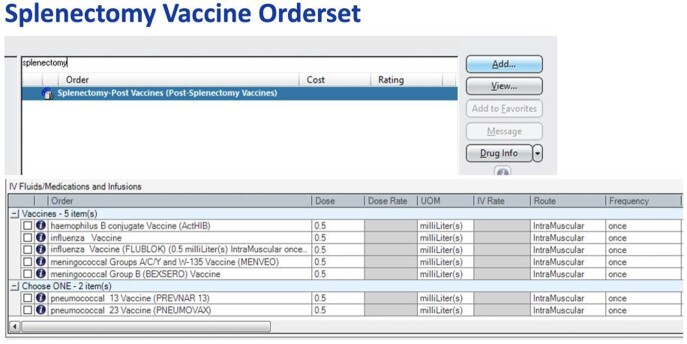

An orderset was created to mitigate errors with vaccine selection for patients undergoing splenectomy.

**Disclosures:**

**All Authors**: No reported disclosures.

